# Relationship Between Muscle Thickness and Hip and Groin Function in Elite Professional Dancers: A Cross-Sectional Correlational Study

**DOI:** 10.3390/jcm15176554

**Published:** 2026-08-25

**Authors:** Miguel A. Alcocer-Ojeda, Antonia Gómez-Conesa

**Affiliations:** 1 International School of Doctoral Studies, University of Murcia, Campus Espinardo, 30100 Murcia, Spain; miguelangel.alcocer@um.es; 2 Research Group Methods and Evaluation in Social Sciences Regional Campus of International Excellence (CEIR) Mare Nostrum Campus (CMN), University of Murcia, 30100 Murcia, Spain

**Keywords:** dance, hip, groin, pain, skeletal muscles, ultrasonography

## Abstract

**Objective**: The aim of the study was to sonographically evaluate the thickness of the abdominal, multifidus lumborum and gluteal muscles in professional dancers and the relationship between anthropometric, demographic and professional characteristics and clinical tests of the hip and groin. **Methods**: A cross-sectional study was employed, examining ultrasound imaging in 20 elite professional ballet dancers to assess the thickness of the abdominal muscles, lumbar multifidus, gluteus medius and gluteus minimus. The associations between the ultrasound variables and hip and groin function were tested using the Copenhagen Hip and Groin Outcome Score (HAGOS) and the Beighton score. The Spearman correlation coefficient was used to find the relationship between muscle thickness, Beighton score and HAGOS. **Results**: The increase in muscle thickness from rest to activity was greater in the transversus abdominis left (62.6%) and the internal oblique left (38.7%). The strongest correlations between muscle thickness and HAGOS were observed for the gluteus medius left at rest (0.55) and active (0.58) in Physical Function in Sport and Recreation and right active in Symptoms (0.42), Physical Function in Sport and Recreation (0.48), and Participation in Physical Activities (0.54). The correlation of the Beighton score with muscle thickness was inverse for the internal oblique left at rest (−0.50) and active (−0.48), the transversus abdominis active left (−0.52) and right (−0.56), the rectus abdominis right at rest (−0.55), and the gluteus minimus right at rest (−0.51). **Conclusions**: The muscle thickness of the gluteus medius was positively correlated with the HAGOS questionnaire, whereas joint hypermobility was negatively correlated with internal oblique, transversus abdominis, rectus abdominis and gluteus minimus muscle thickness. Ultrasound imaging should be considered for the measurement of muscle thickness in hip and groin pain. **Trial Registration**: ClinicalTrials.gov, NCT0565416. Date of registration: 8 December 2022.

## 1. Introduction

In pre-professional dancers, 47% report a history of injury [1], with injury rates of 1.06 and 1.46 per 1000 h of dance for men and women, respectively [2]. These injuries primarily affect the lower limbs. Hip and groin injuries are an important health issue for dancers of all skill levels, with an injury rate of 17.7% (14.1% in students and 27.6% in professional dancers) [3]. Additionally, amateur dancers suffer more frequently from hip/groin/thigh injuries [4].

In professional ballet dancers, the incidence of musculoskeletal injuries ranges from 47.1% to 72.6% per season. Specifically, 75.6% report lower limb injuries per season, with the most frequently affected regions being the foot (49.3%) and ankle (45.1%) [5].

Lower extremity injuries comprise 66% to 91% of all injuries in professional ballet dancers, with foot and ankle accounting for 14% to 57% of these. The main cause of injuries is overuse (64% of female injuries and 50% of male injuries). The prevalence of hip pain reported from ballet dancers is 62% in the lumbosacral region, 29% in the patellofemoral region, and 58% for painful snapping hip [6].

Groin pain (GP) may be due to extra-articular anatomical structures, such as the tendons of the adductor muscles [7], psoas [8], fascia latae [8], and gluteal muscles [9] or intra-articular structures, such as injuries to the labrum [10]. The prevalence of labral tears, identified by magnetic resonance imaging, has been found in 51% of 196 hips, with no differences between ballet and sports professionals [9].

In view of the high training load, at an average of 27.1 h per week, professional dancers are considered elite athletes [3]. In athletes with hip and/or groin pain, decreased hip and abdominal muscle thickness has been found [11]. Furthermore, decreased strength in the muscles of the abdomen and gluteal region has been documented [12]. In addition, using electromyographic recordings, a delay in transversus abdominis (TrA) activation during the active straight leg raise test has been observed in athletes with prolonged GP [11].

Ultrasound studies conducted in dancers have shown greater TrA thickness at rest, and greater TrA and lumbar multifidus thickness increase during contraction [13]. Moreover, dancing requires an extreme range of motion of the hips, and dancers exhibit greater strength of hip external rotation in the final range of motion; the research involving ballet dancers utilizing external rotation has revealed that their deep external hip rotator muscles are not larger than those of non-dancer athletes [14]. Variations in muscle thickness across the lumbopelvic–hip complex, including the TrA, internal oblique (IO), lumbar multifidus, gluteus medius (GM), and gluteus minimus (GMN), are closely tied to joint stability, force transmission, and self-reported hip groin functionality [15]. Also, strengthening with electrical muscle stimulation and strength training produces changes in resting abdominal, gluteal, and hip adductor muscle thickness on ultrasound in non-athletic adults. The resting muscle thickness did not differ between the groups; however, the contracted muscle thickness was higher with electrical muscle stimulation [16].

Although ballet dancers perform using external rotation, magnetic resonance imaging has found that their deep hip external rotator muscles are no larger than those of other athletes [12]. Despite the critical importance of evaluating muscle thickness in dancers, whether for diagnostic or therapeutic purposes, to the best of our knowledge, no studies to date have examined changes in the thickness of abdominal and gluteal muscles in dancers with and without GP.

The purpose of this study was to sonographically evaluate abdominal muscle thickness, lumbar multifidus, GM and GMN, and to determine their possible relationship with anthropometric, demographic and professional characteristics, as well as clinical and functional hip and groin tests in professional ballet dancers. Our hypothesis was that in the sample of dancers, the increase in muscle thickness from rest to activity would be greater in the transversus abdominis, rectus abdominis and internal oblique. We also hypothesized that reduced muscle thickness would be associated with higher joint hypermobility. Additionally, we hypothesized that both age and years of dance experience would have an influence on hip or groin pain.

## 2. Materials and Methods

### 2.1. Study Design

A cross-sectional study was conducted, registered with ClinicalTrials.gov, Clinical Trials Identifier: NCT05654168 (date of registration: 8 December 2022), and approved by the ethics committee for clinical research of the University of Murcia, Murcia, Spain, 2585/2019. The recommendations established in the STROBE (Strengthening the Reporting of Observational Studies in Epidemiology) guide were followed [17].

### 2.2. Population, Sample and Data Collection

The study was conducted on dancers at the National Ballet of Spain (*Ballet Nacional de España*), whose choreographies combine elements of classical ballet and Spanish dance. At the time of the study, the total population of elite professional dancers from the National Ballet of Spain was 33. Before the study began, the study was presented personally to the dancers, and it was also explained that the data obtained would be used for scientific purposes. Although all gave their written consent to participate in the study, 13 dancers declined to participate in the ultrasound imaging examination due to fear of infection during the COVID-19 pandemic, which had surpassed 12 million cases in Spain by May 2022 (with a population of around 47.7 million).

Finally, 20 dancers (10 women and 10 men) participated in the study. Beyond a personal clinical report at the end of the study, no incentives were provided to the participants.

Data collection included participant characteristics and assessments using the following measurement tools: the Beighton score (joint hypermobility) [18]; the HAGOS (Copenhagen Hip and Groin Outcome Score) questionnaire (hip or groin pain) [19]; and the measurement of abdominal muscle thickness, lumbar multifidus, GM and GMN using ultrasound imaging (USI). All measurements were performed under standardized conditions by the same trained physiotherapist with 25 years of clinical experience.

### 2.3. HAGOS Questionnaire

This questionnaire consists of six self-reported subscales assessing Pain, Symptoms, Physical Function in Daily Living, Physical Function in Sport and Recreation, Participation in Physical Activities, and hip and/or groin-related Quality of Life (QOL). For each of the subscales, a score of 0 represented extreme hip and/or groin problems, and a score of 100 represented no hip and/or groin problems. The test–retest reliability with intra-class correlation coefficients (ICC) ranged from 0.82 to 0.91 for the six subscales [19].

### 2.4. Beighton Score

This score was used to measure joint hypermobility, which is classified as a score of ≥4. The Beighton score assesses five joint movements and is interpreted based on the maximum score of 9. This test has shown excellent reliability (ICC = 0.992; 95% confidence interval, 0.979–0.997) [20].

### 2.5. USI Measurements

Previous studies have shown that the intra- and inter-rater reliability of USI is good to excellent for the measurement of the thickness of the muscles under study: TrA, IO, external oblique (EO) and GM (ICC = 0.82 to 0.92) [21]; ICC = 0.990 to 0.99 and 0.995 to 0.999 [22]; TrA, IO, EO and rectus abdominis (RA) (ICC = 0.77 to 0.89) [23]; and TrA and lumbar multifidus (ICC = 0.88 to 0.94 and 0.80 to 0.92) [24].

### 2.6. USI Examination Procedures

A Toshiba Nemio XG ultrasound machine was used in B-mode, and muscle thickness was measured using electronic calipers of an ultrasound unit multi-frequency convex probe, 3–6 MHz. Measurements were taken of the thickness of the muscle groups on the right and left sides according to the best available evidence, and two ultrasound images were taken of each muscle, one at rest and the other during contraction. For the examination of abdominal muscles, the dancer was in a supine position with the knees extended, and the transverse probe was above the iliac crest in the midaxillary line at the level of the navel and outside the RA. At the end of an exhalation, after several normal breathing cycles, the image was frozen. Resting thickness was measured from the inferior fascial edge to the superior fascial edge of each of the lateral muscles of the abdomen [25]. For the RA, the probe was positioned at the umbilical level until the image of the muscle was visualized, and the resting thickness was measured in the central part of the muscle belly following the same procedure [26]. To measure the thickness during muscle contraction, the dancer was asked to raise their right leg approximately 5 cm towards the hip joint (neutral hip position, both internal rotation–external rotation and abduction–adduction); the image was frozen after 10 s to measure the layer thickness of the TrA, IO, EO and RA [27].

For the measurement of the thickness of the lumbar multifidus muscle, the dancer was in a prone position; the arms were positioned above the head with 120 degrees abduction and 90 degrees of elbow joint flexion, and longitudinal images were obtained at the L4–L5 level with a maximum depth of 10 cm at rest and during contralateral arm elevation [28].

Finally, for the GMN and GM muscles, the dancer was in a side-lying position with the hip in neutral flexion, extension and 20 degrees of adduction (inclinometer confirmed). The convex probe was placed longitudinally on the lateral side of the hip, cranially between the upper part of the greater trochanter and the iliac crest, and at a point 25% of the distance from the latter, between the anterior superior and posterior superior iliac spines. After adjusting the craniocaudal position of the transducer until the upper lip of the acetabulum and the head of the femur appeared, the thickness was measured at rest and during hip abduction in the central part of the muscle belly of the GMN and GM [29]. Once the dancer was positioned, the pressure biofeedback unit was inflated so that the starting position was 20 degrees of hip adduction from the horizontal, and they were instructed to gently lift their leg toward the ceiling with their toes pointing forward, retaining this position until the reading on the pressure biofeedback unit decreased by 20 mmHg.

The image processing and analysis was performed by the same evaluator, with 13 years’ experience performing USI examinations daily.

**Figure 1:**
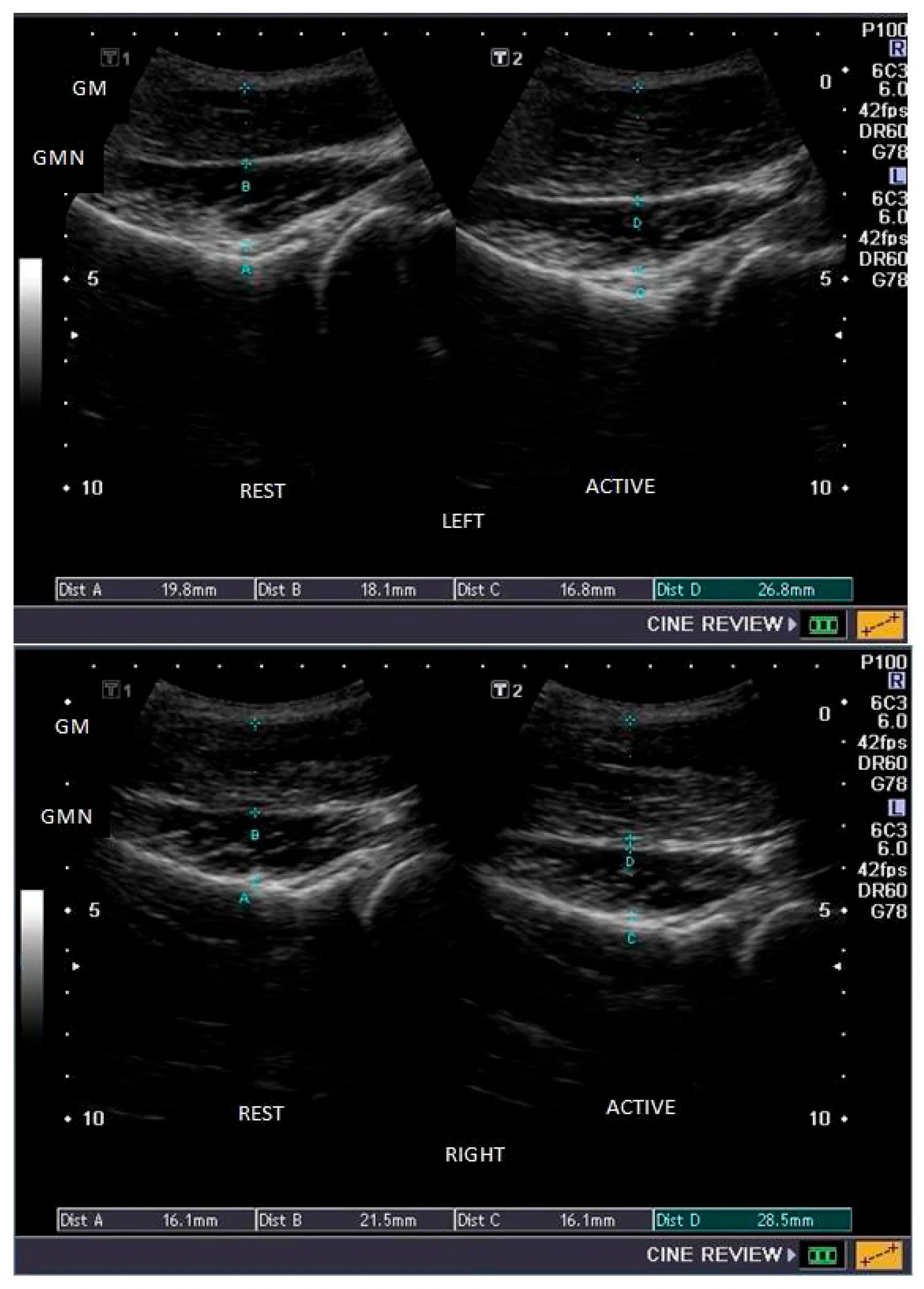
Measurement of the gluteus medius (GM) and gluteus minimus (GMN) muscles at rest and active, right side and left side.

**Figure 2:**
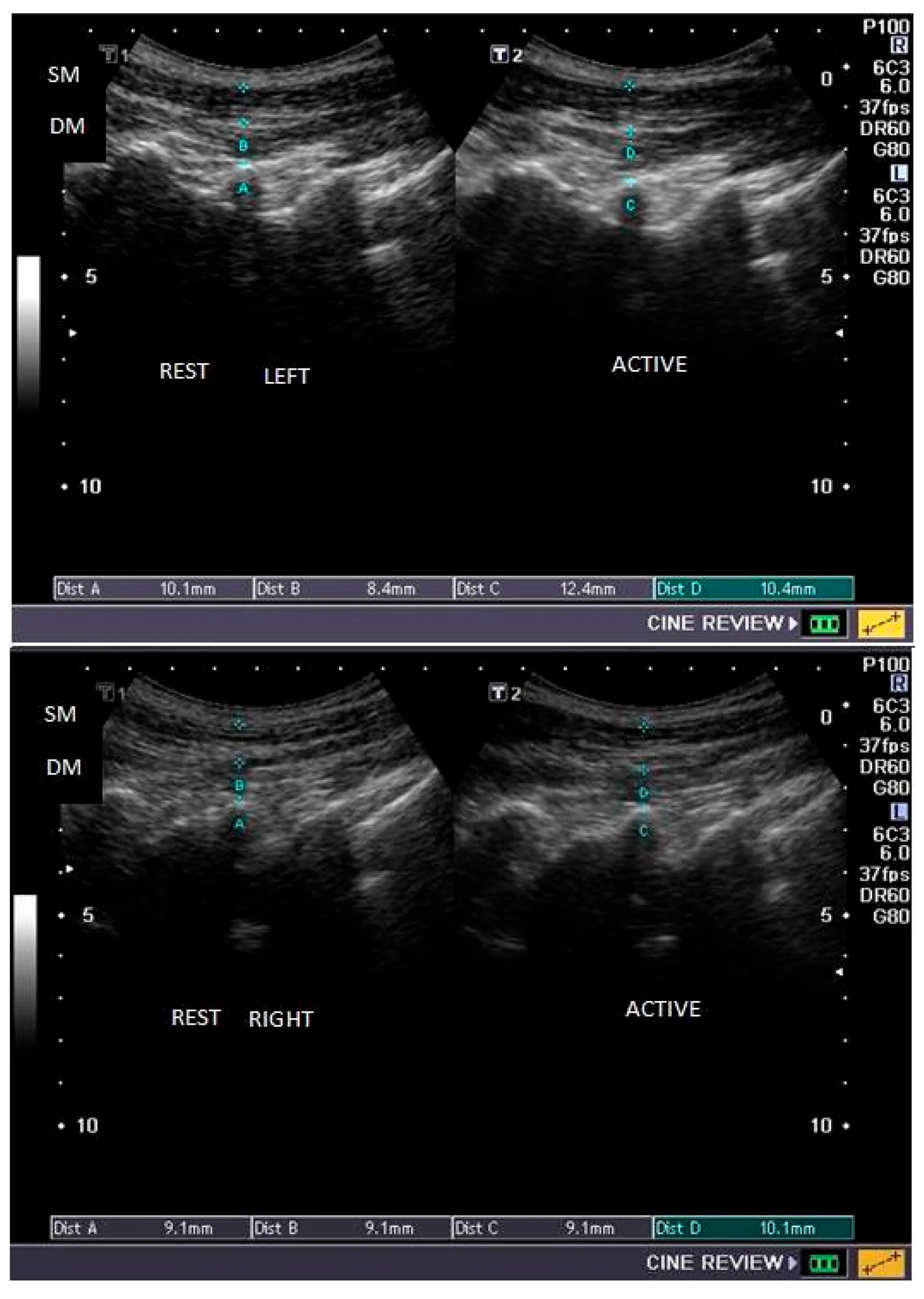
Measurement of the deep multifidus (DM) and superficial multifidus (SM) muscles at rest and active, left side and right side.

The measurement procedures for the GM, GMN, deep multifidus (DM) and superficial multifidus (SM) muscles, at rest and active, on the right and left sides, are illustrated in Figure 1 and Figure 2.

To assess inter-rater reliability, an inter-rater reliability analysis was carried out prior to participant evaluation. The muscle thicknesses of the GM, GMN, DM and SM were measured by two physiotherapists (both experienced raters, one of whom was the evaluator in the present study). Measurements were performed simultaneously on five healthy subjects, three women and two men, aged between 22 and 35, weighing from 50.5 to 66 kg, and with a height from 160 to 179 cm, with a Body Mass Index (BMI) from 20.6 to 24.8, considered a healthy BMI. All of them were amateur athletes. The results (intra-class correlation coefficients, Standard Error of Measurements, and minimal detectable changes) are shown in Table 1.

### 2.7. Statistical Analysis

Statistical analyses were performed with Microsoft Excel 2021 (Microsoft Corp., Redmond, WA, USA) and JAMOVI v. 2.3.13. Intra-class correlation coefficients were used to verify the inter-rater reliability of the ultrasound imaging. Participant characteristics, HAGOS, Beighton score and muscle thickness are shown using means, standard deviations, and minimum and maximum values of each of the measures. Using the Kolmogorov–Smirnov test with a 95% confidence interval and significance level of 0.05, the normal distribution of variables was tested. The results show that the age, height and weight variables follow a normal distribution, whereas the HAGOS questionnaire, Beighton scale and muscle thickness, both at rest and active, do not follow a normal distribution. Muscle thicknesses by sex were compared using Mann–Whitney U tests and were presented using the median and interquartile range. A *p*-value of <0.05 was considered statistically significant. Also, effect sizes (rank–biserial correlation coefficient = *r*) were calculated and interpreted as small (<0.30), medium (0.30–0.50), or large effects (>0.50).

The Spearman correlation coefficient (r), with a 95% confidence level and significance level of 0.05, was used to calculate the correlation between muscle thickness, the Beighton scale and HAGOSs. Spearman’s correlation coefficient was interpreted as: weak = 0.1 to 0.3; moderate = 0.3 to 0.5; strong = 0.5 to 0.7; or very strong = 0.7–1.

The person conducting the data analysis was blinded at all times.

## 3. Results

The participant characteristics are presented in Table 2.

### 3.1. HAGOS Questionnaire

The pain subscale mean score was 73.9. Considering that a score of 100 indicates no problems, and 0 indicates extreme problems, our participants had a score slightly below the third quartile. The Symptoms and QOL subscales obtained the lowest values, 65.2 and 65.5, respectively. In the remaining subscales of the HAGOS questionnaire, the average scores were above 75 (Table 2).

### 3.2. Beighton Score

The Beighton score yielded a mean value of 4.9. Considering that a positive Beighton score requires 4 points or more, our sample of professional dancers had joint hypermobility (Table 2).

### 3.3. Muscle Thickness

Table 3 shows muscle thickness at rest and during activity, and Table 4 shows a comparison of muscle thickness by sex. Muscle thickness was higher in men than in women, with a significant difference (*p* < 0.05) in RA and IO at rest bilaterally (effect size: 0.69 left and 0.44 right). A significant difference was found (*p* = 0.019) with greater thickness in females in EO active left (effect size 0.46), as shown in Table 3 and Table 4.

**Figure 3:**
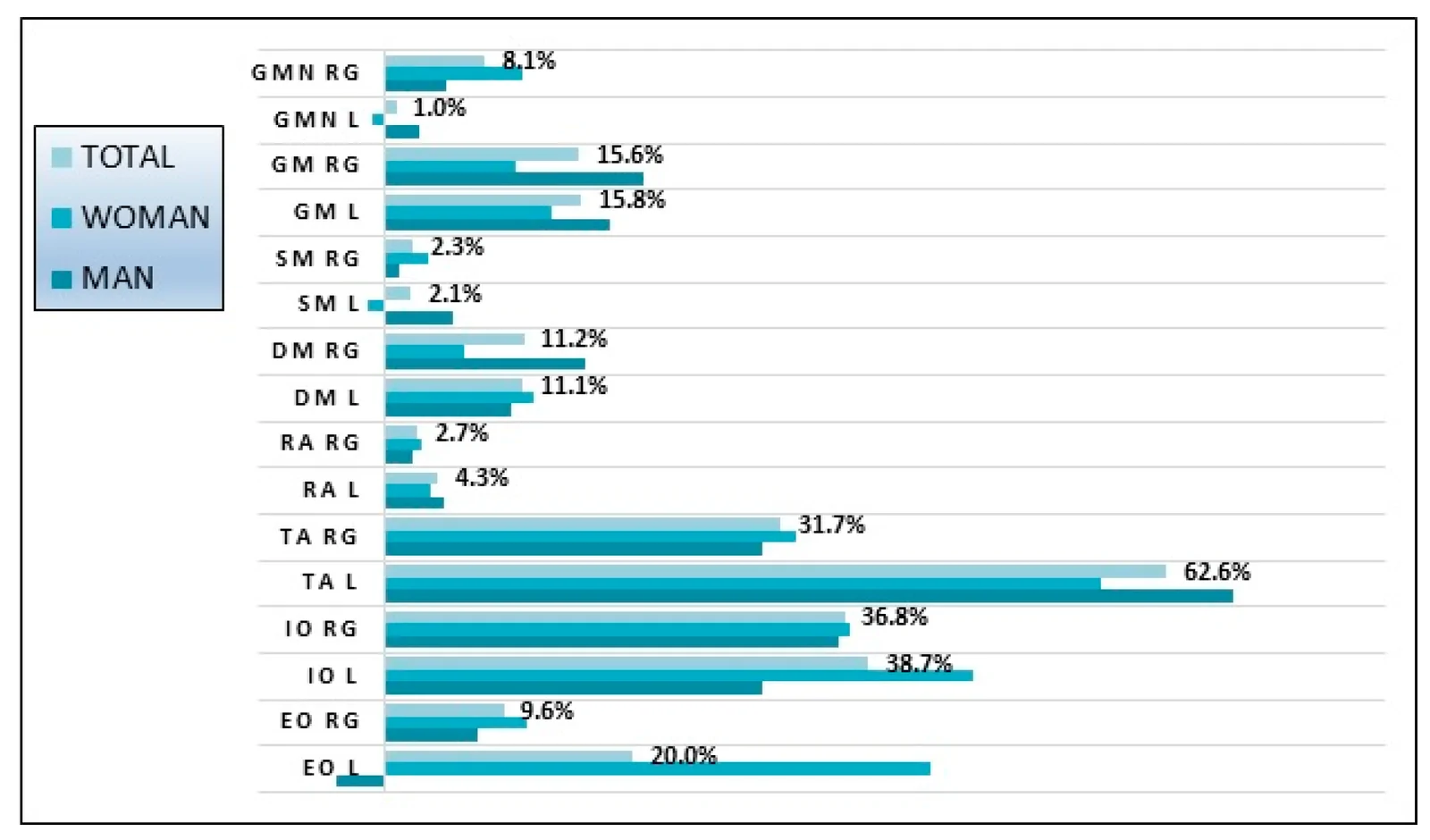
Variation in muscle thickness in percentage from rest to activity (n = 20).

Also, Figure 3 displays the % change in thickness between rest and activity (Figure 3).

### 3.4. Abdominal Muscles

The muscle with the greatest increase in thickness between rest and active was the TrA, with a mean increase of 0.99 mm (31.7%) on the right and 1.88 mm (62.6%) on the left side. For the IO, the mean increase was 2.68 mm (36.8%) for the right and 2.45 mm (38.7%) for the left. The % increase in thickness in the EO and RA was lower than in the TrA and IO.

### 3.5. Lumbar Multifidus

The mean muscle thickness increase in the right DM from rest to contralateral arm elevation was 1.40 mm (11.2%), compared to 1.40 mm in the left DM (11.1%). In the SM, the increase was 2.1% on the left side and 2.3% on the right.

### 3.6. Gluteal Muscles

The GM revealed an increase of 15.8% on the left side and 15.6% on the right side. In the GMN, the increase on the left side was 1%, compared to 8.1% on the right side.

### 3.7. Correlations Between Muscle Thicknesses, Beighton Score and HAGOS

Table 5 shows correlations greater than 0.3 between participant characteristics, muscle thicknesses, Beighton score and HAGOS, and the confidence interval (CI) (Table 5). In addition, all correlations and the CI are presented in Supplementary Table S1.

In the correlation of the Beighton scale with the characteristics of the participants, BMI stands out with a moderate inverse correlation (−0.46). Regarding the correlation between participant characteristics and the HAGOS questionnaire, both age and years of dance practice showed a strong inverse correlation (−0.53 and −0.68, respectively) with the pain subscale score.

These two characteristics also show a moderate–strong inverse correlation with the HAGOS subscales, except for Participation in Physical Activities.

The correlation between muscle thickness and the Beighton scale reveals a moderate to strong inverse correlation with the abdominal musculature, and a moderate inverse correlation with the lumbar multifidus musculature. Considering the gluteal musculature, a strong inverse correlation with the Beighton score was found (−0.51) with the thickness of the GMN at rest right, and a moderate positive correlation (0.43) with the thickness of the GM active left.

The correlation between abdominal muscle thickness and the scores of the HAGOS questionnaire is generally weak, except for TrA at rest right with QOL (−0.36). Considering the gluteal musculature based on the subscales of the HAGOS questionnaire, there is a strong correlation with the muscle thickness of the GM active right and the subscale Physical Function in Sport and Recreation (0.54), the GM active left with pain (0.55), and the DM rest left with Physical Function in Daily Living (0.54).

## 4. Discussion

USI has been a good alternative for visualizing normal and pathological skeletal muscles and for non-invasively measuring parameters that reflect muscle size [30]. Consequently, studies have been conducted to validate USI for measuring muscle and determine its test–retest reliability. Studies comparing USI with magnetic resonance imaging, which is more costly and often inaccessible, have demonstrated excellent validity for assessing muscle size, with ICC values ranging from 0.998 to 0.999 [31]. Following the first studies using USI to measure muscle cross-sectional areas, its use in clinical practice, research, and sports has increased, and other USI parameters have subsequently been incorporated into assessments of muscle characteristics. Muscle thickness has been used to assess recovery from muscle damage after exercise [32] and to monitor the effects of resistance training on muscle size and strength [33,34].

This exploratory cross-sectional study aimed to sonographically evaluate the thickness of abdominal muscles, lumbar multifidus, GM, and GMN in elite professional ballet dancers and examine their bivariate associations with joint hypermobility (Beighton score) and self-reported hip and groin clinical outcomes (HAGOS questionnaire).

Of the 20 participants in the study, 16 self-reported hip and/or groin pain, representing 80% of the sample. This proportion is higher than the 27.6% reported by Trentacosta et al. [3] in professional ballet dancers, who had fewer hours of weekly training than our participants (27.1 vs. 33.8) and a lower mean age (17.9 years vs. 32.5 years). Considering that the incidence rate of hip and/or groin injuries in previous studies has been reported as 0.09 per 1000 h of dancing [3], age, hours of training, and dance experience (mean, 15.3 years) could contribute to the higher prevalence of hip and/or groin pain in our sample. Additionally, the pain subscale of the HAGOS questionnaire showed a strong inverse correlation with age (−0.53) and an even stronger inverse correlation with years of dance experience (−0.68). In our study, dancers who were older and had more years of dance experience were more likely to report hip- and/or groin-related clinical symptoms. These findings align with the initial hypotheses; however, due to the cross-sectional design of this study, these correlations should be interpreted as non-causal.

Qamar et al. [35] noted that the prevalence of joint hypermobility may vary across age groups, emphasizing the relevance of considering BMI as a potential influencing factor in joint hypermobility. These authors observed that hyperextension of the right knee was associated with lower BMI.

We found a moderate inverse correlation (−0.46) between BMI and the Beighton score, indicating that lower BMI was associated with greater joint hypermobility in our sample. Additionally, we observed a moderate positive correlation (0.32) between the Beighton score and the pain subscale of the HAGOS questionnaire. Previous studies have yielded contradictory findings in this regard; Mayes et al. [36] found no relationship between the two variables, whereas Scott et al. [37] pointed out that hypermobility is well documented in dancers and has been proposed as a potential contributor to hip microinstability. They also found that Beighton scores were higher in dancers with hip pain than in symptomatic non-dancers. Naal et al. [38] reported a 33% prevalence of ligament hyperlaxity in people with GP, although this was associated with radiologically diagnosed femoroacetabular impingement syndrome. In our sample, these anatomical alterations were not investigated.

Consistent with research on elite sportspeople, muscle thickness was greater in men than in women in our study, with significant differences in RA both at rest and during activation and in IO at rest on both the left and right sides. Male elite alpine skiers generally have thicker core muscles than female skiers on both sides [39].

In physically fit, active soldiers, Teyhen et al. [40] found that males had thicker muscles than females in IO at rest and during activation, with greater muscle cross-sectional area for the RA and multifidus (since cross-sectional area does not allow differentiation between DM and SM). They also reported greater muscle thickness at rest and during activation in males, with increases of 10.49% in IO, 9.27% in TrA and 23.38% in the multifidus. The increases from rest to activation were higher in our study: right TrA (31.7%), left TrA (62.6%), right IO (36.8%) and left IO (38.7%), and similar to those reported in the adult population by Cuellar et al. [41], with increases in right TrA (50.66%), left TrA (63.53%), right IO (30.54%), and left IO (37.08%). These findings largely align with our hypotheses regarding increases in muscle thickness.

Stokes et al. [28] found a larger multifidus in men than in women and a smaller multifidus in patients with chronic pain than in asymptomatic patients based on cross-sectional area measurements (without differentiating between DM and SM). The cross-sectional area of the multifidus decreases in dancers with low back pain and/or hip pain [42], and is lower in athletes with severe hip, groin, or thigh injuries during the preseason [43]. In our study, left DM at rest showed moderate to strong correlations with several HAGOS subscales (Symptoms, 0.38; Physical Function in Sport and Recreation, 0.38; Participation in Physical Activities, 0.46; and Physical Function in Daily Living, 0.54).

Despite the changes in muscle thickness between rest and the straight leg raise of the TrA (62.6% left and 31.7% right) and IO (38.7% left and 36.8% right), the correlations between abdominal muscle thickness and the HAGOS subscales were weak. In teenage male soccer players, Jansen et al. [44] found decreased left TrA thickness at rest in players with prolonged GP. Linek et al. [45] found moderate to strong correlations between IO thickness at rest and the Physical Function in Daily Living (0.40), Physical Function in Sport and Recreation (0.57), and QOL (0.41) HAGOS subscales. They also found a moderate correlation between TrA thickness at rest and the QOL subscale (0.47).

Although certain measurements in our study, such as resting DM thickness, showed correlations with HAGOS subscales ranging from r = 0.32 to 0.46, their 95% confidence intervals included zero. Consequently, these findings should be viewed strictly as preliminary, exploratory trends characterized by high uncertainty rather than as confirmed functional associations.

Regarding the correlation between abdominal muscle thickness and the Beighton score, we found moderate to strong inverse correlations, between the Beighton score and right RA at rest (−0.55), right RA during activation (−0.37), left RA at rest (−0.45), right IO at rest (−0.46), right IO during activation (−0.48), left IO at rest (−0.50), left IO during activation (−0.48), right TrA at rest (−0.34), right TrA during activation (−0.56), and left TrA during activation (−0.52). Previous studies of adults with hyperlaxity have found partially impaired function of the lateral abdominal muscles when attempting to increase TrA thickness [46] and impaired contraction of TrA [47]. In our sample, which was characterized by joint hypermobility (mean Beighton score, 4.9), greater TrA thickness and greater thickness of the lateral abdominal musculature were found. The negative correlation between the Beighton score and TrA thickness suggests that dancers with greater hypermobility may exhibit reduced muscle thickness or impaired activation capacity; however, causality cannot be established, and the relationship may be influenced by confounding factors such as sex, age, and training history.

The USI procedures used in our study to measure muscle thickness were based on the best available evidence, and reliability was good to excellent for the muscles under study. For examination of the abdominal muscles, the dancer was in a supine position, whereas the GMN and GM were examined in a side-lying position.

Given that TrA and GM play important roles in stabilizing the lumbopelvic–hip complex, the reliability of muscle thickness measurements during standing and single-leg standing has also recently been evaluated. The inter-rater reliability of TrA at rest in asymptomatic subjects was ICC 0.86 (95% confidence interval, 0.48–0.96) during standing and ICC 0.95 (95% confidence interval, 0.80–0.98) during single-leg standing; the inter-rater reliability of GM at rest was ICC 0.99 (95% confidence interval, 0.83–0.99) during standing and ICC 0.98 (95% confidence interval, 0.84–0.99) during single-leg standing [48]. In the lying position, the inter-rater reliability of TrA at rest in asymptomatic subjects was ICC 0.83 (95% confidence interval, 0.63–0.92) [49]. In addition, the inter-rater reliability of GM in a side-lying position was ICC 0.85 (95% confidence interval, 0.62–0.94) [50].

GM can be more difficult to assess than other muscles due to participant positioning and probe placement [50]. In our study, the inter-rater reliability of right GM at rest prior to the study was ICC 0.99 (95% confidence interval, 0.99–1.00) and was considered excellent.

We found an increase in muscle thickness from rest to activation in right GM (15.6%), left GM (15.8%), right GMN (8.1%), and left GMN (1%), with no significant differences between men and women. Farley et al. [22] reported GM thickness measurements of 31.1 mm at rest and 29.3 mm during activation, with a 1.8% change in thickness, also assessed in the side-lying position. On the other hand, Whiler et al. [51] found no relationship between GM and GMN muscle thickness and lower extremity muscle strength in adults. In our study, GM and GMN thickness were assessed at rest and during hip abduction; it is possible that a loaded position would have resulted in greater activation [52].

McMillan et al. [53] noted that, compared with athletes of the same age, sex and weight, professional ballet dancers have greater cross-sectional area at the GM and greater GMN muscle thickness. They also reported that hip pain was not related to the size of the gluteal musculature. In contrast, Perraton et al. [54] found, using magnetic resonance imaging, that active young adults with longstanding hip and/or groin pain, regardless of sex, had larger lateral hip muscle volumes and greater hip strength. In our study, we found moderate positive correlations between the pain subscale score and right GM thickness during activation (0.42) and left GM thickness during activation (0.55). There were also moderate positive correlations between right and left GM thickness during activation and all HAGOS subscales (except Participation in Physical Activities). However, in a recent meta-analysis assessing the occurrence of groin pain, 1516 healthy athletes were followed up (97% male), of whom 478 experienced at least one episode of groin pain. Athletes who remained healthy displayed greater hip adduction strength, whereas no differences in hip abduction strength were observed between injured and uninjured athletes, neither overall nor among athletes with groin injuries [55].

Ultrasound examination can be considered a first-choice method for the functional diagnosis of the hip [56]. In this study, USI enabled us to evaluate muscle thickness in professional ballet dancers and establish relationships with hip and/or groin clinical symptoms. Future research and clinical use of USI could provide new insights into the functional assessment of muscle thickness and GP among professional dancers.

## 5. Limitations

The current study has several limitations. First, the cross-sectional design limits our ability to infer causality or temporality from the observed relationships, and no adjustment for potential confounders was performed. Second, only dancers from the National Ballet of Spain were included, which may limit the generalizability of our results to dancers of different ages and with varying years of experience. In addition, our sample of 20 elite professional dancers represents only 60.61% of the population of the National Ballet of Spain. Likewise, the relationship between BMI and the Beighton score may be confounded by sex and should therefore be interpreted with caution, as sex was not included as a covariate in the analyses, which may have resulted in residual confounding.

Although the exploratory analyses in this study yielded promising findings for future confirmation, an important limitation is that no corrections for multiple comparisons were performed.

Additionally, although all USI measurements were carried out by an experienced researcher, the inter-rater reliability study was performed only for thickness measurements of four muscles.

## 6. Conclusions

Our study provides important insights into hip and groin muscle thickness, both at rest and during activation, as well as its association with hip and/or groin function in professional ballet dancers. Our results revealed that abdominal muscle thickness was not correlated with HAGOS subscales, whereas multifidus thickness was correlated with several subscales (Symptoms, Physical Function in Sport and Recreation, Physical Function in Daily Living and Participation in Physical Activities). In addition, bilateral GM thickness during activation and right GM thickness during activation were positively correlated with HAGOS subscales, whereas joint hypermobility was not correlated with HAGOS subscales. From a practical point of view, USI should be considered for measuring hip and groin muscle thickness.

## Figures and Tables

**Table 1:** Intra-class correlation coefficients, Standard Error of Measurements, and minimal detectable change in the muscle thickness measurements.

Muscle	ICC (95% CI)	SEM	MDC
GM right rest	0.999 (0.994–1.000)	0.0922	0.256
GM right active	1.000 (0.998–1.000)	0.0592	0.164
GMN left rest	0.999 (0.997–1.000)	0.0632	0.175
GMN left active	1.000 (0.978–0.998)	0.0548	0.152
DM right rest	1.000 (1.000–1.000)	0	0.0
DM right active	1.000 (1.000–1.000)	0	0.0
SM left rest	1.000 (1.000–1.000)	0	0.0
SM left active	1.000 (1.000–1.000)	0	0.0

CI, confidence Interval; DM, deep multifidus; GM, gluteus medius; GMN, gluteus minimus; ICC, intra-class correlation coefficient; MDC, minimal detectable change; SEM, Standard Error of Measurements; SM, superficial multifidus.

**Table 2:** Participant characteristics, HAGOS and Beighton score. Values provided include the mean; SD and minimum and maximum values; and median and interquartile range. (n = 20).

Participant Characteristics	Mean ± SD and (Min–Max)
Age (years)	32.5 ± 5.8 (22–44)
Weight (Kg)	61.2 ± 6.9 (49–71)
Size (cm)	170.1 ± 7.6 (156–180)
Body Mass Index	21.1± (18.6–24.3)
Dance starting age	7 ± 2.6 (3–13)
Dance training per week (hours)	33.8 ± 5.3 (15–40)
Dance experience (years)	15.3 ± 5.8 (6–29)
**HAGOS (range 0–100)**	**Median (IQR)**
Pain	81.3 (25.6)
Symptoms	67.9 (17.0)
Function in activities of daily living	92.5 (17.5)
Function in sport and recreation	84.4 (31.3)
Participation in physical activities	75.0 (37.5)
Quality of life	70 (28.8)
**Beighton score (range 0–9)**	5.0 (2.3)

HAGOS, Copenhagen Hip and Groin Outcome Score; IQR, interquartile range; Min–Max, minimum and maximum values; SD, standard deviation.

**Table 3:** Muscle thickness measured in mm at rest and during activity. Values provided include the median and interquartile range (IQR). (n = 20).

Muscles	Left Rest Median (IQR)	Left Active Median (IQR)	Right Rest Median (IQR)	Right Active Median (IQR)
EO	3.9 (1.0)	4.25 (1.60)	3.60 (1.0)	3.80 (1.70)
IO	6.40 (1.6)	8.65 (3.30)	7.10 (2.3)	8.80 (5.13)
TrA	2.70 (0.6)	4.35 (1.38)	3.10 (0.6)	3.55 (1.98)
RA	9.85 (2.3)	10.40 (2.68)	9.95 (2.1)	9.95 (1.93)
DM	13.60 (4.2)	15.26 (3.88)	13.25 (2.9)	13.95 (4.33)
SM	9.70 (1.9)	9.90 (1.18)	9.40 (2.3)	9.90 (1.63)
GMN	17.45 (2.6)	17.10 (1.25)	17.10 (3.2)	17.80 (3.85)
GM	24.20 (3.2)	27.0 (3.63)	23.50 (3.0)	26.85 (5.18)

DM, deep multifidus; EO, external oblique; GM, gluteus medius; GMN, gluteus minimus; IO, internal oblique; RA, rectus abdominis; RIC, interquartile range; SM, superficial multifidus; TrA, transversus abdominis.

**Table 4:** Muscle thickness measured in mm at rest and while active. Values provided include the median in women (n = 10) and men (n = 10).

Muscle	Rest Men	Rest Women	*p*-Value	Dif (95% CI)	Effect size = *r*	Active Men	Active Women	*p*-Value	Dif (95% CI)	Effect Size = r
EO L	4.1	3.4	0.385	−0.65 (−1.5–0.2)	0.194	3.8	4.85	0.140	0.75 (−0.30–2.30)	**0.330**
EO RG	3.40	3.6	0.838	0.20 (−0.70–1.60)	0.047	3.80	3.70	0.705	−0.15 (−1.40–0.90)	0.085
IO L	7.6	6	* 0.002	−1.95 (−3.30–−0.70)	**0.693**	8.65	7.40	0.186	−1.10 (−3.80–1.10)	0.296
IO RG	7.8	5.9	* 0.006	−1.90 (−3.30–−0.80)	**0.628**	9.70	7.30	0.131	−2.20 (−5.40–0.60)	**0.338**
TrA L	3	2.70	0.273	−0.20 (−0.80–0.20)	0.245	4.15	3.80	0.406	−0.50 (−1.50–0.80)	0.186
TrA RG	3.10	2.60	* 0.037	−0.40 (−0.90–−0.10)	**0.478**	3.80	3.10	0.345	−0.60 (−2.10–0.80)	0.211
RA L	10.7	9.05	* 0.028	−1.70 (−3.30–−0.20)	**0.490**	10.80	8.70	0.076	−1.80 (−3.60–0.10)	**0.397**
RA RG	10.7	8.95	* 0.041	−1.55 (−3.10–−0.10)	**0.456**	11.0	9.30	0.076	−1.70 (−3.30–0.20)	**0.397**
DM L	14.6	11.6	* 0.028	−3.10 (−5.70–−0.40)	**0.490**	15.95	12.75	* 0.045	−3.10 (−6.10–−0.10)	**0.448**
DM RG	14.2	11.9	0.064	−1.90 (−3.70–0.30)	**0.414**	14.60	11.95	* 0.026	−2.70 (−5.30–−0.30)	**0.499**
SM L	9.7	9.05	0.910	−0.65 (−1.70–0.70)	0.025	10.10	9.40	0.186	−0.70 (−2.00–0.60)	0.296
SM RG	9.75	9.25	0.678	−0.40 (−2.00–1.40)	0.093	9.70	10.10	0.821	0.35 (−1.30–1.60)	0.051
GM L	24.2	23.8	0.940	−0.35 (−3.40–2.70)	0.017	26.80	27.35	0.650	0.80 (−2.70–4.20)	0.101
GM RG	23.5	22.1	0.427	−1.0 (−4.50–2.0)	0.177	28.70	25.55	0.121	−3.45 (−8.0–0.80)	**0.347**
GMN L	16.3	17.4	0.705	0.85 (−2.10–3.80)	0.085	16.95	17.30	0.791	0.30 (−1.40–2.40)	0.059
GMN RG	17.1	16.7	0.910	−0.30 (−2.70–2.30)	0.025	17.80	17.80	0.496	0.30 (−2.70–3.80)	0.152

CI, confidence interval; Dif, difference between groups; DM, deep multifidus; EO, external oblique; GM, gluteus medius; GMN, gluteus minimus; IO, internal oblique; L, Left; Min–Max, minimum and maximum values; RA, rectus abdominis; RG, right; SM, superficial multifidus; TrA, transversus abdominis. Mann–Whitney test with a significance level at * *p* < 0.05. Effect size (rank–biserial correlation = r). r greater than or equal to 0.3 (medium–large effect) is given in boldface.

**Table 5:** Spearman correlation coefficient and confidence interval (95%) between participant characteristics, muscle thicknesses, Beighton score and HAGOS.

	Beighton Score	HAGOS PAIN	HAGOS SYMPTOMS	HAGOS ADL	HAGOS SPORT	HAGOS PA	HAGOS QOL
Beighton	-	**0.32** (−0.14–0.67)					
BMI	**−0.46** **(−0.75–0.02)**						
Age (years)		**−0.53** **(−0.80–0.10)**	**−0.52** **(−0.79–0.09)**	**−0.32** (−0.67–0.14)	**−0.44** (−0.74–0.01)		**−0.51** **(−0.78–−0.08**)
Dance experience (years)		**−0.68** **(−0.87–0.33)**	**−0.61** **(−0.84–0.23)**		**−0.48** **(−0.76–0.05)**		**−0.58** **(−0.82–−0.18)**
**Muscle Left Rest** IO	**−0.50** **(−0.77–0.07)**						
RA	**−0.45** **(−0.75–0.01)**						
DM	**−0.30** (−0.65–0.16)		**0.38** (−0.07–0.70)	**0.54** **(0.12–0.80)**	**0.38** (−0.07–0.70)	**0.46** **(0.02–0.75)**	
GM		**0.32** (−0.14–0.67)	**0.34** (−0.12–0.68)		**0.55** **(0.14–0.80)**		**0.35** (−0.11–0.68)
**Left Active** EO		**0.31** (−0.15–0.66)		**0.49** **(0.1–0.8)**			
IO	**−0.48** **(−0.8–−0.0)**						
TrA	**−0.52** **(−0.8–−0.1)**						
SM	**−0.39** (−0.71–0.06)			**0.30** (−0.16–0.65)		**0.49** **(0.1–0.8)**	
GM	**0.43** (−0.02–0.74)	**0.55** **(0.1–0.8)**	**0.40** (−0.05–0.71)	**0.39** (−0.06–0.71)	**0.58** **(0.2–0.8)**		**0.39** (−0.06–0.71)
**Muscle Right Rest** IO	**−0.46** **(−0.7–−0.0)**						
TrA	**−0.34** (−0.68 0.12)						**−0.36** **(−0.7–0.1)**
RA	**−0.55** **(−0.8–−0.1)**			**0.34** (−0.12–0.68)			
SM	**−0.33** (−0.67–0.13)			**0.51** **(0.1–0.8)**			
GMN	**−0.51** **(−0.8–−0.1)**						**−0.31** (−0.67–0.15)
GM				**0.34** (−0.12–0.68)			
**Right Active** IO	**−0.48** **(−0.8–−0.0)**						
TrA	**−0.56** **(−0.8–−0.2)**						
RA	**−0.37** (−0.70–0.08)			**0.30** (−0.16–0.65)			
DM	**−0.41** (−0.72–0.05)			**0.34** (−0.12–0.68)	**0.30** (−0.16–0.65)		
SM				**0.37** (−0.08–0.70)			
GMN				**0.34** (−0.12–0.68)			
GM		**0.42** **(0.0–0.7)**	**0.35** (−0.11–0.68)	**0.48** **(0.1–0.8)**	**0.54** **(0.1–0.8)**		**0.39** (−0.06–0.71)

ADL, Physical Function in Daily Living; BMI, Body Mass Index; Dance experience, years of experience; DM, deep multífidus; EO, external oblique; GM, gluteus medius; GMN, gluteus minimus; IO, internal oblique; PA, Participation in Physical Activities; QOL, Quality of Life; RA, rectus abdominis; SM, superficial multifidus; SPORT, Physical Function in Sport and Recreation; TrA, transversus abdominis.

## Data Availability

All data relevant to the study are available upon reasonable request to the corresponding author.

## Supplementary Materials

The following supporting information can be downloaded at https://www.mdpi.com/article/10.3390/jcm15176554/s1. Table S1. Spearman correlation coefficient and confidence interval (95%) between muscle thicknesses, Beighton score and HAGOS.
