# Supplementary material for: Relationship Between Muscle Thickness and Hip and Groin Function in Elite Professional Dancers: A Cross-Sectional Correlational Study
Source: J Clin Med. 2026 Aug 25;15(17):6554. doi: 10.3390/jcm15176554 (PMC13566605; doi:10.3390/jcm15176554)
Supplement: Supplementary file 1 [file jcm-15-06554-s001.zip › jcm-4440133-supplementary.pdf]

Table S1. Spearman correlation coefficient and confidence interval (95%) between muscle thicknesses, Beighton and HAGOS scores.

Muscle	Beighton Score	HAGOS PAIN	HAGOS SYMPTOMS	HAGOS ADL	HAGOS SPORT	HAGOS PA	HAGOS QOL
**Left Rest**							
EO	-0.04	-0.06	0.06	**0.34**	0.14	0.13	0.00
	(-0.47 - 0.41)	(-0.49 - 0.39)	(-0.40 - 0.49)	(-0.12 - 0.68)	(-0.32 - 0.55)	(-0.33 - 0.54)	(-0.44 - 0.44)
IO	**-0.50**	-0.20	-0.21	0.14	-0.15	0.02	-0.02
	**(-0.77- -0.07)**	(-0.59 - 0.26)	(-0.60 - 0.25)	(-0.32 - 0.55)	(-0.56 - 0.3)]	(-0.43 - 0.46)	(-0.46 - 0.43)
TrA	-0.22	-0.20	-0.12	0.23	0.11	0.06	0.02
	(-0.60 - 0.24)	(-0.59 - 0.26)	(-0.53 - 0.34)	(-0.23 - 0.60)	(-0.35 - 0.52)	(-0.40 - 0.49)	(-0.43 - 0.46)
RA	**-0.45**	0.12	0.13	0.27	0.09	0.22	-0.03
	**(-0.75- -0.01)**	(-0.34 - 0.53)	(-0.33 - 0.54)	(-0.19 - 0.63	(-0.37 - 0.51)	(-0.24 - 0.60)	(-0.46 - 0.42)
DM	**-0.30**	0.16	**0.38**	**0.54**	**0.38**	**0.46**	0.28
	(-0.65 - 0.16)	(-0.30 - 0.56)	(-0.07 - 0.70)	**(0.12 - 0.80)**	(-0.07 - 0.70)	**(0.02 - 0.75)**	(-0.18 - 0.64)
SM	-0.11	-0.15	-0.03	0.16	0.02	0.27	-0.05
	(-0.52 - 0.35)	(-0.56 - 0.31)	(-0.46 - 0.42)	(-0.30 - 0.56)	(-0.43 - 0.46)	(-0.19 - 0.63)	(-0.48 - 0.40)

GMN	-0.15	0.15	0.08	0.15	-0.09	0.22	-0.01
	(-0.56 - 0.31)	(-0.31 - 0.56)	(-0.38 - 0.50)	(-0.31 – 0.56)	(-0.51 - 0.37)	(-0.24 - 0.60)	(-0.45 - 0.44)
GM	0.21	**0.32**	**0.34**	0.27	**0.55**	0.15	**0.35**
	(-0.25 - 0.59)	(-0.14 - 0.67)	(-0.12 - 0.68)	(-0.19 - 0.63)	**(0.14 - 0.80)**	(-0.31 - 0.56)	(-0.11 - 0.68)
**Left Active**							
EO	0.09	**0.31**	0.27	**0.49**	0.24	0.24	0.15
	(-0.37 - 0.51)	(-0.15 - 0.66)	(-0.19 - 0.63)	**(0.1 - 0.8)**	(-0.22 - 0.61)	(-0.22 - 0.61)	(-0.31 - 0.56
IO	**-0.48**	0.21	0.21	**0.33**	0.18	0.02	0.02
	**(-0.8 - -0.0)**	(-0.25 - 0.59)	(-0.25 - 0.59)	(-0.13 - 0.67)	(-0.28 - 0.58)	(-0.43 - 0.46)	(-0.43 - 0.46)
TrA	**-0.52**	0.04	0.16	0.25	-0.03	0.19	-0.23
	**(-0.8 - -0.1)**	(-0.41 - 0.48)	(-0.30 - 0.56)	(-0.21 - 0.62)	(-0.46 - 0.42)	(-0.28 - 0.58)	(-0.61 - 0.23)
RA	-0.26	0.16	0.05	0.18	-0.03	0.12	-0.19
	(-0.63 - 0.20)	(-0.30 - 0.56)	(-0.40 - 0.49)	(-0.28 - 0.58)	(-0.46 - 0.42)	(-0.34 - 0.53)	(-0.59 - 0.27)
DM	-0.26	-0.05	0.15	0.38	0.13	0.26	-0.05
	(-0.63 - 0.20)	(-0.48 - 0.40)	(-0.31 - 0.56)	(-0.07 - 0.70)	(-0.33 - 0.54)	(-0.20 - 0.63)	(-0.48 - 0.40)
SM	**-0.39**	0.08	0.24	**0.30**	0.03	**0.49**	-0.14
	(-0.71 - 0.06)	(-0.38 - 0.50)	(-0.22 - 0.61)	(-0.16 - 0.65)	(-0.43 - 0.46)	**(0.1 - 0.8)**	(-0.55 - 0.32)

GMN	-0.28	-0.16	-0.03	0.11	-.025	0.15	-0.27
	(-0.64 - 0.18)	(-0.56 - 0.30)	(-0.46 - 0.42)	(-0.35 - 0.52)	(-0.62 - 0.21)	(-0.31 - 0.56)	(-0.63 - 0.19)
GM	**0.43**	**0.55**	**0.40**	**0.39**	**0.58**	0.19	**0.39**
	(-0.02 - 0.74)	**(0.1 - 0.8)**	(-0.05 - 0.71)	(-0.06 - 0.71)	**(0.2 - 0.8)**	(-0.28 - 0.58)	(-0.06 - 0.71)
**Right Rest**							
EO	0.02	-0.15	-0.26	0.08	-0.14	0.20	0.03
	(-0.43 - 0.46)	(-0.56 - 0.31)	(-0.63 - 0.20)	(-0.38 - 0.50)	(-0.55 - 0.32)	(-0.27 - 0.59)	(-0.43 - 0.46)
IO	**-0.46**	-0.01	0.01	0.26	0.07	0.04	-0.02
	**(-0.7 - -0.0)**	(-0.45 - 0.44)	(-0.44 - 0.45)	(-0.20 - 0.63)	(-0.39 - 0.49)	(-0.41 - 0.48)	(-0.46 - 0.43)
TrA	**-0.34**	-0.28	-0.29	-0.16	-0.20	0.19	**-0.36**
	(-0.68 - 0.12)	(-0.64 - 0.18)	(-0.64 - 0.17)	(-0.56 - 0.30)	(-0.59 - 0.26)	(-0.28 - 0.58)	**(-0.7- 0.1]**
RA	**-0.55**	0.12	0.13	**0.34**	0.20	0.22	0.12
	**(-0.8 - -0.1)**	(-0.34 - 0.53)	(-0.33 - 0.54)	(-0.12 - 0.68)	(-0.27 - 0.59)	(-0.24 - 0.60)	(-0.34 - 0.53)
DM	-0.18	-0.17	0.09	0.29	0.29	0.28	0.21
	(-0.58 - 0.28)	(-0.57 - 0.29)	(-0.37 - 0.51)	(-0.17 - 0.64)	(-0.17 - 0.64)	(-0.18 - 0.64)	(-0.25 - 0.59)

SM	**-0.33**	0.16	0.27	**0.51**	0.17	0.21	0.01
	(-0.67 - 0.13)	(-0.30 - 0.56)	(-0.19 - 0.63)	**(0.1 - 0.8)**	(-0.30 - 0.56)	(-0.25 - 0.59)	(-0.44 - 0.45)
GMN	**-0.51**	-0.13	0.04	0.13	-0.26	0.19	**-0.31**
	**(-0.8 - -0.1)**	(-0.54 - 0.33)	(-0.41 - 0.48)	(-0.33 - 0.54)	(-0.63 - 0.20)	(-0.28 - 0.58)	(-0.67 - 0.15)
GM	0.20	0.04	-0.03	**0.34**	0.20	0.17	0.18
	(-0.27 - 0.59)	(-0.41 - 0.48)	(-0.46 - 0.42)	(-0.12 - 0.68)	(-0.27 - 0.59)	(-0.30 - 0.56)	(-0.28 - 0.58)
**Right Active**							
EO	0.00	0.01	-0.17	0.28	-0.02	0.13	-0.01
	(-0.44 - 0.44)	(-0.44 - 0.45)	(-0.57 - 0.29)	(-0.18 - 0.64)	(-0.46 - 0.43)	(-0.33 - 0.54)	(-0.45 - 0.44)
IO	**-0.48**	0.07	0.02	0.20	-0.03	-0.07	-0.20
	**(-0.8 - -0.0)**	(-0.39 - 0.49)	(-0.43 - 0.46)	(-0.27 - 0.59)	(-0.46 - 0.42)	(-0.49 - 0.39)	(-0.59 - 0.26)
TrA	**-0.56**	0.08	0.13	0.20	0.00	0.06	-0.18
	**(-0.8 - -0.2)**	(-0.38 - 0.50)	(-0.33 - 0.54)	(-0.27 - 0.59)	(-0.44 - 0.44)	(-0.40 - 0.49)	(-0.58 - 0.28)
RA	**-0.37**	0.16	0.10	**0.30**	0.12	0.17	0.01
	(-0.70 - 0.08)	(-0.30 - 0.56)	(-0.36 - 0.51)	(-0.16 - 0.65)	(-0.34 - 0.53)	(-0.30 - 0.56)	(-0.44 - 0.45)
DM	**-0.41**	-0.11	0.18	**0.34**	**0.30**	0.19	0.15
	(-0.72 - 0.05)	(-0.52 - 0.35)	(-0.28 - 0.58)	(-0.12 - 0.68)	(-0.16 - 0.65)	(-0.28 - 0.58)	(-0.31 - 0.56)

SM	-0.23	-0.01	0.07	**0.37**	0.09	0.22	0.01
	(-0.60 - 0.24)	(-0.45 - 0.44)	(-0.39 - 0.49)	(-0.08 - 0.70)	(-0.37 - 0.51)	(-0.24 - 0.60)	(-0.44 - 0.45)
GMN	-0.27	-0.01	0.16	**0.34**	-0.06	0.20	-0.16
	(-0.63 - 0 .20)	(-0.45 - 0.44)	(-0.30 - 0.56)	(-0.12 - 0.68)	(-0.49 - 0.39)	(-0.27 - 0.59)	(-0.56 - 0.30)
GM	0.19	**0.42**	**0.35**	**0.48**	**0.54**	0.28	**0.39**
	(-0.28 - 0.58)	**(0.0 - 0.7)**	(-0.11 - 0.68)	**(0.1 - 0.8)**	**(0.1 - 0.8)**	(-0.18 - 0.64)	(-0.06 - 0.71)

ADL, Physical Function in Daily Living; DM, Deep Multífidus; EO, External Oblique; GM, Gluteus Medius; GMN, Gluteus Minimus; IO, Internal Oblique; PA, Participation in Physical Activities; QOL, Quality of Life; RA, Rectus Abdominis; SM, Superficial Multifidus; SPORT, Physical function in Sport and Recreation; TrA, Transversus Abdominis.

Spearman correlation coefficient (r) are interpreted as: weak = 0.1 to 0.3; moderate= 0.3 to 0.5; strong= 0.5 to 0.7; very strong = 0.7-1.0. Values r greater than 0.3 (moderate correlation) are in bold.

If Confidence interval (CI) does not include the value 0, the association is statistically significant. CI does not include 0 are in bold.
